# Isolated Exercise Interventions for Acute Low Back Pain: Systematic Review and Meta-Analysis of Randomized Controlled Trials

**DOI:** 10.3390/healthcare13172209

**Published:** 2025-09-03

**Authors:** Melania Cardellat-González, Luis González-Gómez, Juan-David Guzmán-Gómez, Laura Blanco-Heras, Andrés Arana-Rodríguez, Álvaro-José Rodríguez-Domínguez

**Affiliations:** 1 Department of Health and Sports, Pablo de Olavide University, 41009 Seville, Spain; melaniac.2697@gmail.com; 2 Department of Nursing and Physiotherapy, University of Cadiz, 11009 Cadiz, Spain; 3 Department of Surgery and Medical-Surgical Specialties, University of Oviedo, 33003 Oviedo, Spain; 4 Department of Physiotherapy, University of Seville, 41092 Seville, Spain; 5 Department of Nursing, University of Huelva, 21004 Huelva, Spain

**Keywords:** back pain, acute low back pain, therapeutic exercise, exercise therapy, conservative treatment

## Abstract

**Background**: Therapeutic exercise (TE) is recommended as the first line of treatment for low back pain (LBP), but questions remain about the true efficacy of TE in the acute phase. This study aimed to evaluate the effectiveness of isolated TE in reducing pain intensity and disability in patients with acute or subacute LBP. **Methods**: A systematic review with meta-analysis was conducted following the PRISMA guidelines. Randomized controlled trials (RCTs) that analyzed therapeutic exercise alone in one of the intervention groups and assessed pain intensity and disability were included; both outcomes were considered primary in this review. The quality of evidence was assessed using the GRADE tool. **Results**: Five RCTs were included. Meta-analyses were performed in subgroups according to the comparators: usual care, education, manual therapy, and bed rest. Statistically significant differences in favor of TE were found only when compared to usual care (SMD = −0.23; 95% CI [−0.45, −0.01]; *p* = 0.04). **Conclusions**: TE, when prescribed as an isolated intervention, appears to be more effective than usual care in improving short-term disability outcomes in patients with acute LBP. However, the limited quality and number of available studies, together with the typically favorable natural course of acute LBP, suggest that these findings should be interpreted with caution. Current evidence supports the integration of exercise within a comprehensive, multimodal management plan that addresses the physical, psychological, and social dimensions of pain.

## 1. Introduction

Low back pain (LBP) is one of the most prevalent health problems worldwide and a leading contributor to socioeconomic burden. In 2020, more than 619 million people were affected globally and, although age-standardized rates have shown a slight decline over the past three decades, projections suggest this number will exceed 800 million by 2050 [1]. In addition to its high prevalence, LBP is the leading cause of years lived with disability worldwide, leading to substantial economic burdens through increased healthcare costs, reduced productivity, and decreased quality of life for those affected [2].

Most consultations for low back pain (LBP) involve acute or subacute episodes, defined as lasting less than four weeks and between four and twelve weeks, respectively, and are predominantly classified as nonspecific, meaning no identifiable pathoanatomical cause can be determined [3,4]. In these cases, pain is primarily due to peripheral and central sensitization of nociceptors, along with alterations in pain modulation and psychosocial factors [5].

Although acute LBP typically has a favorable prognosis, with spontaneous symptom resolution within the first six weeks [6], it is estimated that between 25% and 50% of patients experience recurrences or progress to chronic forms [7]. This transition to recurrent or chronic LBP is associated with a significant increase in healthcare and societal costs, along with functional and psychosocial deterioration that severely impacts the autonomy and well-being of those affected [8].

In recent decades, the clinical approach to acute and subacute LBP has shifted toward patient-centered models that recognize the biopsychosocial nature of pain [9]. International clinical practice guidelines recommend first-line interventions such as educating patients about the origin of their pain and strategies for self-management, maintaining usual physical activity, the use of oral or topical analgesics or anti-inflammatory drugs only when necessary, and the early implementation of active therapeutic strategies [10,11].

In this context, physiotherapy plays a fundamental role, with therapeutic exercise (TE) serving as a key intervention for both symptom control and the prevention of chronicity [12]. This strategy encompasses a wide range of modalities, including aerobic, strengthening, and mobility exercises, among others [13], supported by multiple mechanisms of action. From a biomechanical perspective, it enhances strength and motor control [14]; psychologically, it reduces kinesiophobia and promotes active pain coping strategies [15,16]; and, neurophysiologically, it activates descending inhibitory pathways and stimulates endorphin release, thereby contributing to its hypoalgesic effect [17,18].

Although it is included as a first-line intervention in clinical practice guidelines, questions remain regarding the true effectiveness of TE in the early stages of LBP [19]. The scientific literature presents mixed findings concerning its impact on pain and disability in patients with acute or subacute LBP, hindering the formulation of clear and consistent clinical recommendations [20]. Moreover, existing reviews have assessed the effectiveness of TE in combination with other treatment modalities [1]; to date, no meta-analysis has evaluated the efficacy of this intervention as a standalone approach. In this context, an updated synthesis of the available evidence is warranted to assess the specific impact of TE in this population and to clarify its role in the initial management of LBP.

Therefore, the objective of this study is to conduct a systematic review with meta-analysis of randomized controlled trials (RCTs) to evaluate the effectiveness of an exercise-therapy-based intervention, applied in isolation, for reducing pain intensity and disability in patients with acute or subacute LBP.

## 2. Materials and Methods

### 2.1. Protocol and Registration

A systematic review and meta-analysis of RCTs available in the scientific literature was carried out according to Preferred Reporting Items for Systematic Reviews and Meta-Analyses (PRISMA) guidelines [21]. This study was registered in PROSPERO (International Prospective Register of Systematic Reviews) (CRD420250609523).

### 2.2. Data Sources and Searches

A search was conducted in MEDLINE, Embase, Cochrane Library, Web of Science and Scopus from inception to January 2025. The complete search strategy is presented in Table S1.

Inclusion criteria were defined according to the PICOS framework (Participants, Interventions, Comparisons, Outcomes, and Study Design):

1. Participants: Adults (18–65 years) with acute or subacute LBP of less than 3 months duration.
2. Interventions: Any form of TE or exercise-based programs.
3. Comparisons: Any treatment used as a comparator.
4. Outcomes: pain intensity, functional outcomes, and disability measures.
5. Study design: RCTs.

Both types of low back pain, acute and subacute, were included together in this review, as they are considered part of continuous clinical progression. An “isolated TE intervention” was defined as one in which exercise was the sole therapeutic intervention applied exclusively to the experimental group. Studies that combined exercise therapy with other structured treatments—such as comprehensive educational programs, manual therapy, electrotherapy, or planned pharmacological interventions—were excluded. Studies comparing one exercise modality to another, including participants with pain lasting more than three months, or involving pain of neuropathic origin were excluded. Studies that were not published in English, Spanish, French, Italian, or Portuguese were also excluded.

After an initial screening based on titles and abstracts, a full-text review was conducted for potentially eligible studies. This process was carried out by two independent reviewers (M.-C.G. and L.-G.G.). In case of disagreement, a third reviewer (A.J.-R.D.) was consulted. Studies that met the inclusion criteria were incorporated into the qualitative synthesis and, when possible, included in the meta-analysis.

### 2.3. Data Extraction

Data extraction was performed independently by two authors (M.C.G. and J.D.-G.G.). In the event of disagreement, a third reviewer (A.J.-R.D.) was consulted. Extracted data were entered into a standardized form, including total and group-specific sample sizes, a description of the interventions applied in each group, outcome variables and assessment tools, duration of the intervention and follow-up periods, and reported results in statistical terms (Table 1).

### 2.4. Quality Assessment

Quality assessment was independently performed by two reviewers (M.-C.G. and A.A.-R.). In case of disagreement, a third reviewer (A.J.-R.D.) was consulted.

The methodological quality and risk of bias of the included studies were assessed using the PEDro scale [27]. This tool consists of 11 items and rates methodological quality based on total score: <4 is considered *poor*, 4–5 points is interpreted as *fair*, 6–8 points as *good*, and ≥9 points as *excellent* [28]

The quality of evidence was evaluated by the Grading of Recommendations Assessment, Development and Evaluation (GRADE) using the GRADE Pro/Guideline Development Tool. This tool rates the certainty of evidence as “High,” “Moderate,” “Low,” or “Very Low,” considering factors such as risk of bias, inconsistency, indirectness, imprecision, publication bias, and effect size [29,30].

### 2.5. Data Synthesis and Analysis

Cohen’s Kappa coefficient was used to assess the level of agreement between reviewers during the article selection process. This analysis was performed using Epidat 4.2 software.

A qualitative synthesis (L.G.G. and A.J.-R.D.) was conducted based on the data extracted from the RCTs that met the inclusion criteria. In addition, means and standard deviations were collected to perform a quantitative synthesis (meta-analysis). When studies reported alternative measures of central tendency and dispersion (e.g., median and interquartile range), these were converted using Higgins’ formula [31]. If different outcome measurement tools were used across studies, the standardized mean difference and its standard error were calculated [31].

To assess heterogeneity, the Cochrane thresholds for interpreting the I^2^ statistic were followed: “thresholds for the interpretation of the I^2^ statistic can be misleading, as the importance of inconsistency depends on several factors. A rough guide in the context of meta-analyses of RCTs is as follows: 0% to 40% = might not be important; 30% to 60% = may represent moderate heterogeneity; 50% to 90% = may represent substantial heterogeneity; 75% to 100% = considerable heterogeneity” [31]. Accordingly, a random-effects model was used when I^2^ exceeded 30%; otherwise, a fixed-effects model was applied.

Publication bias was assessed in cases where the meta-analysis included more than 10 studies [32]. Additionally, a sensitivity analysis (Table S1) was performed to evaluate the influence of each individual study on the overall meta-analytic outcome [32]. All statistical analyses were conducted using Review Manager version 5.4.1 software.

### 2.6. Ethical Considerations

This study is a systematic review with meta-analysis that relies solely on data from previously published research. No new data were collected, nor was any personal or identifiable information used in the conduct of this review. Furthermore, all included studies reported having been approved by their respective institutional ethics committees and having obtained informed consent from participants. Therefore, ethical approval was not required for the present study.

## 3. Results

### 3.1. Search Selection

**Figure 1:**
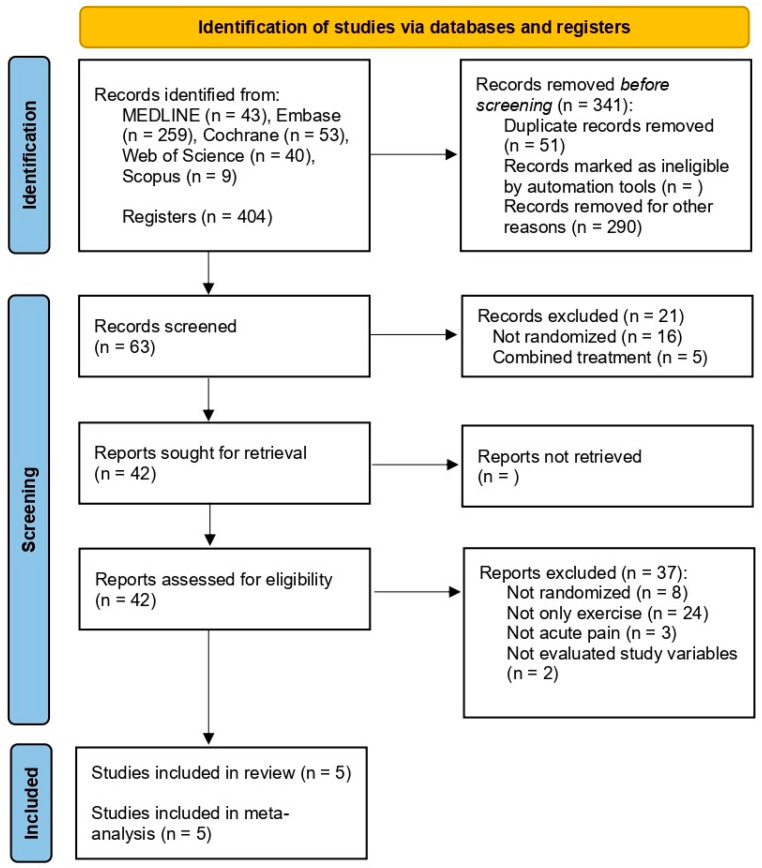
PRISMA flowchart of study selection.

The initial search yielded a total of 404 records. After the screening and eligibility assessment phases, five studies met the inclusion and exclusion criteria and were included in the qualitative synthesis (Table 1). The selection process is detailed in Figure 1.

### 3.2. Characteristics of the Included Studies

All included studies were RCTs published between 1995 and 2006. All were conducted in European countries except for Mayer et al., 2005 [25] and Brennan et al., 2006 [26], which were carried out in the United States.

The total pooled sample consisted of 746 adult patients with acute nonspecific LBP (229 in the intervention groups and 517 in the control groups). The overall attrition rate was 9.52% (71 participants). All included studies explicitly excluded patients with radicular pain, pain of specific/known origin, serious pathologies, neurological disorders, prior spinal surgery, and pregnancy.

The average age of participants was 39 years. Only three studies [24,25,26] reported standard deviations for this variable, with a pooled mean of 39.6 ± 9.9 years. Regarding sex distribution, 64% of participants were women and 36% were men. Detailed characteristics of the included studies are presented in Table 1.

### 3.3. Characteristics of the Interventions: FITT Principle (Frequency, Intensity, Time, and Type)

The most common exercise frequency was 2–3 sessions per week [23,24,26]. One study included an exercise frequency of 5 days per week [25] and another implemented daily sessions until pain remission [22]. The average intervention duration was approximately 8 weeks, ranging from as short as 5 days [25] to protocols lasting up to 15 weeks [24].

The exercise modalities evaluated across the studies included general strength training, specific trunk strengthening exercises (flexion and extension), aerobic exercise programs, and global mobility routines in the intervention groups. Among the control groups, usual care was included, which proved to be heterogeneous across studies and encompassed postural advice, pharmacological guidelines, and application of local heat, among other approaches [22,23,24,25]. Additionally, other comparators included manual therapy [23,26] and cognitive interventions [24]. The mean follow-up duration was approximately 26 weeks.

### 3.4. Methodological Quality of the Included Studies

Overall, the methodological quality of the included studies was rated as “good quality.” Three studies were classified as having “good quality, while two were rated as ‘fair quality.” None of the studies fulfilled items 5 and 6 of the PEDro scale (blinding of participants and therapists); however, this limitation is inherent to the nature of the interventions evaluated (Table 2).

### 3.5. Results of the Meta-Analysis

The meta-analyses for each outcome were stratified by subgroup and categorized based on the type of intervention used in the control groups, which were classified as usual care, education, bed rest, or manual therapy versus TE. All included studies featured three intervention arms, allowing for two pairwise comparisons per study within each meta-analysis (e.g., exercise group vs. control group 1 and exercise group vs. control group 2). In such cases, the sample size of the duplicated control group was halved to avoid inflating the overall effect estimate. One study [25] included four intervention groups; however, the “combined therapy” group was excluded from the analysis, as it involved the use of exercise in conjunction with another intervention.

#### 3.5.1. Results of Pain Intensity

**Figure 2:**
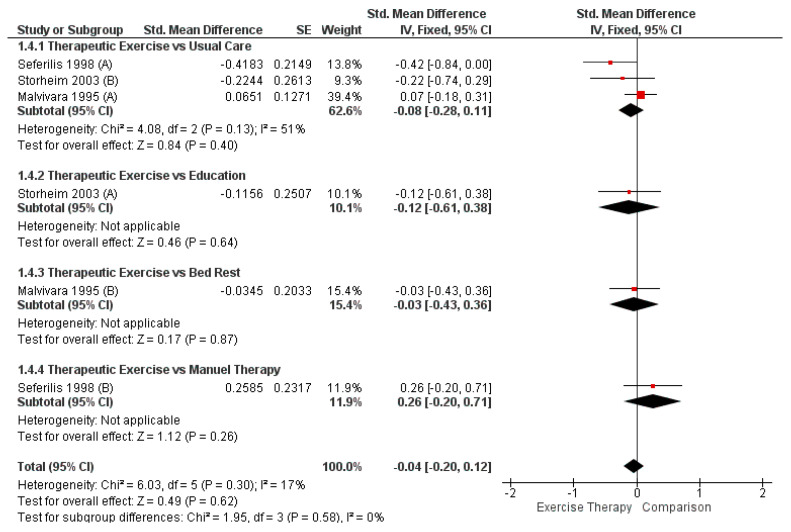
Comparison between therapeutic and control group for pain intensity in acute LBP (forest plot of the meta-analysis [22,23,24]).

Four of the five studies [22,23,24,26] included in this review assessed pain intensity using the following tools: Numeric Pain Rating Scale (NPRS) [22,26], Visual Analogue Scale (VAS) [24], and the modified Borg 10-point numerical scale [23] (Table 1). However, one was excluded from the meta-analysis because it did not provide the necessary data [26]. No statistically significant differences were observed in the overall pooled effect (SMD = −0.04; 95% CI [−0.20, 0.12]; *p* = 0.62), nor in any of the subgroup comparisons: TE vs. usual care (SMD = −0.08; 95% CI [−0.28, 0.11]; *p* = 0.40), TE vs. education (SMD = −0.12; 95% CI [−0.61, 0.38]; *p* = 0.64), TE vs. bed rest (SMD = −0.03; 95% CI [−0.43, 0.36]; *p* = 0.87), and TE vs. manual therapy (SMD = 0.26; 95% CI [−0.20, 0.71]; *p* = 0.26). The forest plot for these meta-analyses is presented in Figure 2.

#### 3.5.2. Results of Disability

All studies [22,23,24,25,26] included in this review assessed disability using validated tools: the Oswestry Disability Index (ODI) [22,23,26] and the Roland–Morris Disability Questionnaire (RMDQ) [24,25] (Table 1).

**Figure 3:**
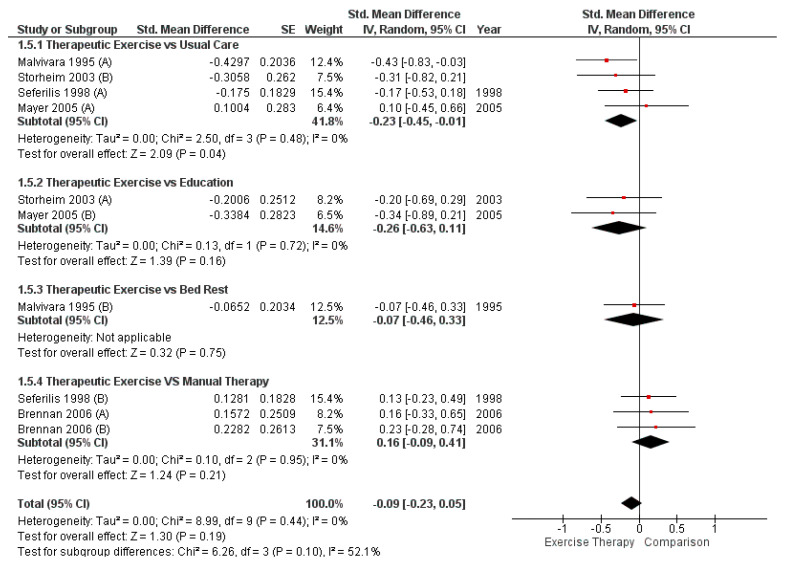
Comparison between TE and control group for disability in acute LBP (forest plot of the meta-analysis [22,23,24,25,26]).

The overall meta-analysis did not reveal statistically significant differences (SMD = −0.09; 95% CI [−0.23, 0.05]; *p* = 0.20). However, a statistically significant difference favoring TE was found in the comparison with usual care (SMD = −0.23; 95% CI [−0.45, −0.01]; *p* = 0.04). No significant differences were observed in the other comparisons: TE vs. education (SMD = −0.26; 95% CI [−0.63, 0.11]; *p* = 0.16), TE vs. bed rest (SMD = −0.07; 95% CI [−0.46, 0.33]; *p* = 0.75), and TE vs. manual therapy (SMD = 0.16; 95% CI [−0.09, 0.41]; *p* = 0.21). The forest plot corresponding to these analyses is shown in Figure 3.

### 3.6. Assessment of the Risk of Publication Bias and Sensitivity Analysis

A minimum of 10 studies is required to reliably assess publication bias, as this analysis has low statistical power with fewer studies and may lead to unreliable results [32]. Therefore, publication bias could not be evaluated. Regarding the sensitivity analysis, no substantial changes in the overall results were observed when each individual study was sequentially removed from the meta-analysis (Table S2).

### 3.7. Synthesis of the Evidence

The strength of the evidence was assessed using the GRADEpro tool. The certainty of the evidence was rated as *very low* for both outcomes analyzed, with their clinical relevance classified as *not important* (Table 3).

## 4. Discussion

The aim of this study was to assess the effectiveness of TE as a standalone intervention for the management of acute LBP, given that all previous reviews to date have evaluated its effectiveness only when combined with other treatment modalities, which makes it difficult to determine the specific effects of exercise. While the management of a multidimensional condition such as pain should not be limited to a single therapeutic approach, it is essential to evaluate each modality independently, as combining interventions may influence their individual effectiveness. Based on our findings and the RCTs currently available, we cannot conclude that TE alone is effective in reducing pain intensity or disability in patients with acute LBP. However, it does appear to be superior to usual care, which typically involves analgesic medication, postural advice, and information about the self-limiting nature of the condition [23].

The improvement in disability observed when compared to usual care—but not to other treatment modalities—may reflect the need to provide specific therapeutic guidance in the context of acute pain. Patients with pain often exhibit kinesiophobia and catastrophic thinking [15], and the provision of structured treatment instructions may enhance coping strategies and self-efficacy [33]. Regarding pain intensity, a recent review of 22 clinical practice guidelines (CPGs) [11] concluded that, although many interventions may be effective in managing acute LBP, the only consistently endorsed recommendations across CPGs are to avoid bed rest and to encourage continued activity. This aligns with the natural course of the condition, as approximately 80% of cases resolve within 4–6 weeks if patients remain active and avoid prolonged rest [34]. However, since around 20% of cases progress to chronicity, it becomes essential to prescribe appropriate treatment and promote physical activity. This represents a significant public health concern, and future efforts in acute pain management should prioritize the prevention of chronicity and recurrence.

Our findings focused on the short-term effects of TE, as few studies included long-term follow-up. For example, Brennan et al. (2006) [26] assessed the effects of manual therapy and two types of TE at both short-term (4 weeks) and long-term (1 year) follow-up. In the short term—relevant to this review—manual therapy showed superior outcomes. However, at the 1-year follow-up, the specific exercise group demonstrated better outcomes than the manual therapy group. Similarly, Storheim et al. (2003) [24] and Malmivaara et al., 1995 [22] evaluated the effects of different TE modalities and control treatments at 12 and 15 weeks, respectively. These time points correspond to post-intervention assessments that fall beyond the acute phase of LBP [34]. This may influence the relative effectiveness of TE, as the observed effects could be partially confounded by the natural course of acute LBP. Measuring outcomes immediately after the intervention, particularly within the acute phase, may provide a more accurate estimate of TE’s specific effects. Consequently, these results should be interpreted with caution, and further research is needed to clarify the short- and long-term impact of these interventions.

To the best of our knowledge, no previous systematic reviews with meta-analysis have specifically evaluated the isolated effect of prescribing TE—without combining it with other treatment modalities—in patients with acute LBP. A recent systematic review with meta-analysis [35] assessed the effectiveness of adding exercise to the treatment of acute LBP. This review included 21 RCTs and concluded that exercise therapy may result in minimal or no differences in pain or disability compared to other interventions. Although consistent with these conclusions, our study exclusively analyzed RCTs in which at least one intervention group received TE as a standalone treatment. As previously discussed, acute LBP tends to follow a natural course of recovery, and many treatments may appear effective simply by coinciding with this recovery trajectory. Supporting this view, a recent network meta-analysis [36] found that both pharmacological and non-pharmacological therapies produced short-term improvements, thereby endorsing non-pharmacological treatments for improving pain intensity and disability in the acute phase. Based on the currently available evidence, clinicians should consider TE as a component of a multimodal intervention that addresses the multidimensional nature of pain, tailored to the patient’s individual characteristics and preferences.

It is also important to consider that the lack of significant benefit versus other active interventions does not imply the absence of clinical value for exercise. Rather, it supports the current paradigm that optimal LBP management should adopt a multimodal, biopsychosocial approach, in which exercise forms one essential component alongside patient education, self-management strategies, and, where appropriate, psychosocial support. The value of exercise in this context may extend beyond direct pain reduction, by promoting movement confidence, counteracting kinesiophobia, and establishing health behaviors that contribute to long-term well-being.

In summary, while the present meta-analysis shows only modest short-term advantages of therapeutic exercise over usual care in acute LBP, it highlights the ongoing need for high-quality research to clarify which patients may benefit most, under what conditions, and as part of what combination of interventions. Clinicians should continue to encourage physical activity, avoid unnecessary rest, and consider exercise as a valuable—but not exclusive—component of a holistic, patient-centered approach.

The findings of our study may have been influenced by several limitations. First, the small number of available studies precludes drawing firm conclusions regarding the effectiveness of isolated TE. Second, the methodological quality of the included trials is inherently limited by the nature of the intervention. RCTs involving exercise therapy cannot feasibly blind participants or therapists, which negatively impacts the overall certainty of the evidence. To avoid overestimating the certainty of evidence due to this limitation, we applied the GRADE tool conservatively in the domain related to risk of bias. Since GRADE also considers other aspects such as inconsistency and imprecision, these factors also contributed to rating the certainty of evidence as very low. Third, most studies assessed only short-term effects, limiting our ability to evaluate the long-term efficacy of TE. Finally, the heterogeneity of the interventions analyzed—regarding the number of sessions, their frequency, total duration, and the types of exercises proposed—precludes strong clinical recommendations, as each study employed a different type of TE. This highlights the need for future RCTs in patients with acute LBP using well-defined and standardized TE protocols, aligned with the existing literature on exercise-induced hypoalgesia [37], as well as outcome measurements that include long-term follow-up. It would also be useful to study the effect of adding exercise therapy to a common base intervention in order to identify potential additional benefits in the treatment of acute low back pain.

## 5. Conclusions

TE, when prescribed as an isolated intervention, appears to be more effective than usual care in improving short-term disability outcomes in patients with acute LBP. That said, no statistically significant differences were found in the remaining disability analyses or in those related to pain intensity. However, the limited quality and number of available studies, together with the typically favorable natural course of acute LBP, require that these findings be interpreted with caution. Current evidence supports the integration of exercise within a comprehensive, multimodal management plan that addresses the physical, psychological, and social dimensions of pain. Future high-quality trials are needed to identify optimal exercise strategies, clarify long-term benefits, and guide individualized treatment for acute LBP. Until then, clinicians are encouraged to combine exercise prescription with education and behavioral support to maximize patient outcomes and prevent chronicity.

## Figures and Tables

**Table 1:** Characteristics of the included studies.

Study	Sample	PEDro Score	Intervention and Follow-Up	Variables Analyzed and Tools	Reported Results
Malmivaara A, 1995 [22]	n = 186 Country: Finland Mean age: 43 years Exercise group: 52 Bed rest group: 67 Usual care group: 67	05/10	Exercise group: Lateral flexion, Back extension *Volume:* 10 rep per exercise *Intensity*: not specified *Frequency*: daily, every two hours *Duration:* 12 weeks Bed rest group: rest completely for 48 h Usual care group: continue with daily activities Follow-up at 3 and 12 weeks (3 months).	Pain Intensity: Numeric Rating Scale (NPRS)/Visual Analog Scale (VAS) Disability: Oswestry Disability Index (ODI)	There were statistically significant results in favor of the active control group and, to a lesser extent, in favor of the Exercise group. Active control group was statistically superior in the acute phase (3 weeks).
Seferlis, 1998 [23]	n = 180 Country: Sweden Mean age: 39 years Exercise group: 60 Manual therapy group: 60 Usual care group: 60	06/10	Exercise group: Intensive general exercises *Volume:* not specified *Intensity*: Phase 1: 20–35% of 1RM (endurance) // Phase 2: 70–95% of 1RM (strength) *Frequency*: 3 per week *Duration*: 8 weeks. Manual Therapy group: at the physiotherapist’s discretion (massage, manipulation, etc.). Average number of sessions: 10 Usual care group: standard treatment (analgesic drugs), advice about posture, and information about the self-curing nature of the disease. Follow-up at 1, 3, and 12 months.	Pain Intensity: Modified Borg Pain Scale (1 “no pain”–11 “maximum”) Pain frequency: Scale 0–8 Disability: Oswestry Disability Index (ODI)	No differences between treatments (natural course of pain).
Storheim, 2003 [24]	n = 93 Country: Norway Mean age: 41 years Exercise group: 30 Education group: 34 Usual care group: 29	06/10	Exercise group: Norwegian aerobic fitness model. *Volume:* 2–3 per week (60 min/session) *Intensity*: Recommendations of the American College of Sports Medicine (ACSM). *Frequency*: 2–3 per week *Duration*: 15 weeks Education group: cognitive intervention about explanation of pain mechanisms and instruction in the squat technique. Usual care group: standard treatment (no restrictions of treatments or referrals). Follow-up at 18 weeks.	Pain Intensity: Visual Analog Scale (VAS) Disability: Roland–Morris Disability Questionnaire (RMDQ)	There were statistically significant improvements in pain reduction for the exercise group. There were significant improvements in disability for the cognitive therapy group.
Mayer, 2005 [25]	n = 100 Country: EEUU Mean age: 31.2 ± 10.6 years Exercise group: 25 Education group: 26 Usual care group: 25 Combined therapy: 24	05/10	Exercise group: Flexion or Back extension according to McKenzie *Volume*: 1–2 sets, 15–20 rep *Intensity:* not specified *Frequency*: Every hour for 5 consecutive days *Duration:* 5 days Education group: patients were given an educational booklet. Usual care group: patients applied a heat wrap (ThermaCare) to the low back region for 8 h/day for 5 consecutive days Combined therapy: heat + exercise. Follow-up at 1, 4 and 7 days	Pain relief: 6-point verbal scale Disability: Roland–Morris Disability Questionnaire (RMDQ) Functional capacity: Multidimensional Task Ability Profile (MTAP)—Rating of Perceived Capacity-Spine (RPC-S)	There were significant improvements in favor of combined therapy group compared to the control group. In terms of functional recovery, the combination therapy group achieved greater complete recovery than the control group. No significant differences were observed between exercise group and heat group.
Brennan, 2006 [26]	n = 123 Country: EEUU Mean age: 37.7 ± 10.7 years Exercise group 1: 34 Exercise group 2: 30 Manual therapy group: 59	07/10	Exercise group 1: Lateral flexion, Back extension exercises *Volume*: not specified *Intensity:* not specified *Frequency*: twice a week *Duration:* 4 weeks Exercise group 2: Trunk strengthening and stabilization exercises *Volume*: not specified *Intensity:* not specified *Frequency*: twice a week *Duration:* 4 weeks Manual therapy group: Thrust manipulation or low amplitude mobilization, twice a week for 4 weeks. Follow-up at 4 weeks and 1 year later	Pain intensity: Numeric Rating Scale (NPRS) Disability: Oswestry Disability Index (ODI)	All groups show improvements in pain and disability

**Table 2:** Summary of results of the PEDro scale.

References	1	2	3	4	5	6	7	8	9	10	11	Total
Malmivaara 1995 [22]	Y	Y	N	N	N	N	Y	Y	N	Y	Y	5/10
Seferlis 1998 [23]	Y	Y	N	Y	N	N	N	Y	Y	Y	Y	6/10
Storheim 2003 [24]	Y	Y	Y	N	N	N	Y	N	Y	Y	Y	6/10
Mayer 2005 [25]	Y	Y	N	Y	N	N	N	Y	N	Y	Y	5/10
Brennan 2006 [26]	Y	N	Y	Y	N	N	Y	Y	Y	Y	Y	7/10

Abbreviations: 1 = eligibility criteria, 2 = random allocation, 3 = concealed allocation, 4 = baseline comparability, 5 = blind subjects, 6 = blind therapists, 7 = blind assessors, 8 = adequate follow-up, 9 = intention-to-treat analysis, 10 = between-group comparisons, 11 = point estimates and variability.

**Table 3:** GRADE. Summary of evidence for pain intensity and disability outcomes according to the GRADE approach, including certainty and clinical importance.

Certainty Assessment							N. of Patients		Effect		Certainty	Importance
N. Studies	Study Design	Risk of Bias	Inconsistency	Indirectly Evidence	Imprecision	Others	I	C	Relative (95% CI)	Absolute (95% CI)		
Pain intensity												
3	RCTs	Serious ^a^	Serious ^b^	Not serious	Serious ^c^	None	95	94	-	SMD −0.04 (−20.0 to 0.12)	⨁◯◯◯ VERY LOW	NOT IMPORTANT
Disability												
5	RCTs	Serious ^a^	Serious ^b^	Not serious	Serious ^c^	None	120	145	-	SMD −0.09 (−0.23 to 0.05)	⨁◯◯◯ VERY LOW	NOT IMPORTANT

Abbreviations: RCTs: randomized controlled trials; I: intervention; C: comparison; CI: confidence interval; SMD: standardized mean difference. ^a^ Heterogeneity may be explained by differences in the type, duration, frequency, or intensity of the interventions applied. ^b^ Variability across studies likely reflects methodological or population differences. Confidence intervals are wide or overlapping, limiting the precision of the estimated effects. ⨁◯◯◯ Indicates the level of certainty.

## Data Availability

Data is contained within the article or Supplementary Materials.

## Supplementary Materials

The following supporting information can be downloaded at: https://www.mdpi.com/article/10.3390/healthcare13172209/s1, Table S1: Search strategy; Table S2: Sensitivity analysis.

## Abbreviations

The following abbreviations are used in this manuscript:

GRADE	Grading of Recommendations Assessment, Development and Evaluation
LBP	Low back pain
PICOS	Participants, Interventions, Comparisons, Outcomes, and Study Design
PRISMA	Preferred Reporting Items for Systematic Reviews and Meta-Analyses
RCTs	Randomized controlled trials
TE	Therapeutic exercise
