# Supplementary material for: Isolated Exercise Interventions for Acute Low Back Pain: Systematic Review and Meta-Analysis of Randomized Controlled Trials
Source: Healthcare (Basel). 2025 Sep 3;13(17):2209. doi: 10.3390/healthcare13172209 (PMC12428032; doi:10.3390/healthcare13172209)
Supplement: Supplementary file 1 [file healthcare-13-02209-s001.zip › healthcare-3747840-supplementary.pdf]

Supplementary material contents

Table S1. Search strategy

Database	Query	Filters
Medline	(“Acute low back pain” OR “Sub-acute low back pain”) AND (“Therapeutic exercise” OR “Exercise program” OR "Exercise" OR "Resistance Training" OR "Strength Training" OR "Aerobic Training" OR "Flexibility Training" OR "Stretching" OR "Aquatic" OR "Pilates" OR "Zumba" OR "Tai Chi" OR "Qigong" OR "Yoga" OR "Mckenzie" OR "Core Stability" OR "Core training" OR "Virtual Reality”) AND (Pain measurement OR pain OR pain assessment OR pain intensity OR functional limitation OR disability OR functionality OR physical function) AND (Randomized clinical trials)	Humans<br><br>RCT
EMBASE	(“Acute low back pain” OR “Sub-acute low back pain”) AND (“Therapeutic exercise” OR “Exercise program” OR "Exercise" OR "Resistance Training" OR "Strength Training" OR "Aerobic Training" OR "Flexibility Training" OR "Stretching" OR "Aquatic" OR "Pilates" OR "Zumba" OR "Tai Chi" OR "Qigong" OR "Yoga" OR "Mckenzie" OR "Core Stability" OR "Core training" OR "Virtual Reality”) AND (Pain measurement OR pain OR pain assessment OR pain intensity OR functional limitation OR disability OR functionality OR physical function) AND (Randomized clinical trials)	RCT
Web of Science	(“Acute low back pain” OR “Sub-acute low back pain”) AND (“Therapeutic exercise” OR “Exercise program” OR "Exercise" OR "Resistance Training" OR "Strength Training" OR "Aerobic Training" OR "Flexibility Training" OR "Stretching" OR "Aquatic" OR "Pilates" OR "Zumba" OR "Tai Chi" OR "Qigong" OR "Yoga" OR "Mckenzie" OR "Core Stability" OR "Core training" OR "Virtual Reality”) AND (Pain measurement OR pain OR pain assessment OR pain intensity OR functional limitation OR disability OR functionality OR physical function) AND (Randomized clinical trials)	RCT
Cochrane Library	(“Acute low back pain” OR “Sub-acute low back pain”) AND ("Therapeutic exercise" OR "Exercise program" OR "Exercise" OR "Resistance Training" OR "Strength Training" OR "Aerobic Training" OR "Flexibility Training" OR "Stretching" OR "Aquatic" OR "Pilates" OR "Zumba" OR "Tai Chi" OR "Qigong" OR "Yoga" OR "Mckenzie" OR "Core Stability" OR "Core training" OR "Virtual Reality") AND (Pain measurement OR pain OR pain assessment OR pain intensity OR functional limitation OR disability OR functionality OR physical function) AND (Randomized clinical trials)	Trials
Scopus	TITLE-ABS-KEY (("Acute low back pain" OR "Sub-acute low back pain") AND ("Therapeutic exercise" OR "Exercise program" OR "Exercise" OR "Resistance Training" OR "Strength Training" OR "Aerobic Training" OR "Flexibility Training" OR "Stretching" OR "Aquatic" OR "Pilates" OR "Zumba" OR "Tai Chi" OR "Qigong" OR "Yoga" OR "Mckenzie" OR "Core Stability" OR "Core training" OR "Virtual Reality") AND (Pain measurement OR pain OR pain assessment OR pain intensity OR functional limitation OR disability OR functionality OR physical function) AND (Randomized clinical trials))	

Table S2. Sensitivity analysis

	Study	TE vs Usual Care											
	CI (95%)	d	IC (95%)		Weight (%)								
Pain intensity	Malvivara 1995 A	0.0569	-0.3377	0.4515	25.46								
	Seferilis 1998 A	-0.4244	-0.8042	-0.0446	27.48								
	Storheim 2003 B	-0.2274	-0.7394	0.2847	15.12								
	Fixed effects	0.0092	-0.19	0.2083									
	**Study**	**TE vs Usual Care**				**TE vs Education**				**TE vs Manual Therapy**			
	**CI (95%)**	**d**	**IC (95%)**		**Weight (%)**	**d**	**IC (95%)**		**Weight (%)**	**d**	**IC (95%)**		**Weight (%)**
Disability	Malvivara 1995 A	0.2192	-0.1765	0.6148	22.21								
	Malvivara 1995 B												
	Mayer 2005 A	0.1020	-0.4527	0.6567	11.30								
	Mayer 2005 B					0.3430	-0.2154	0.9014	43.72				
	Seferilis 1998 A	-0.1765	-0.5528	0.1999	24.56								
	Seferilis 1998 B									0.1290	-0.2402	0.4982	49.98
	Storheim 2003 A					-0.2031	-0.6953	0.2891	56.27				
	Storheim 2003 B	-0.3099	-0.8234	0.2036	13.19								
	Brennan 2006 A									0.1591	-0.3326	0.6508	28.17
	Brennan 2006 B									12.552	0.6968	1.81	21.84
	Fixed effects	-0.0068	-0.1933	0.1797		0.0357	-0.3336	0.4049		0.3835	0.1225	0.6445	

CI: Confidence interval, TE: Therapeutic Exercise.
